# Urinary Volatilomic Profiling Reveals Candidate Metabolomic Signatures Associated with Colorectal Cancer

**DOI:** 10.3390/biology15181578

**Published:** 2026-09-08

**Authors:** Francisca Viveiros, Pedro H. Berenguer, Isabel Jardim, Miguel Camacho, Ana Célia Sousa, Rosa Perestrelo, José S. Câmara

**Affiliations:** 1 CQM-Centro de Química da Madeira, Campus Universitário da Penteada, Universidade da Madeira, 9020-105 Funchal, Portugal; 2109020@student.uma.pt (F.V.); pedro.berenguer@sesaram.pt (P.H.B.); rmp@staff.uma.pt (R.P.); 2 Centro de Investigação Dra. Maria Isabel Mendonça, Hospital Dr. Nélio Mendonça, SESARAM, EPERAM, Avenida Luís de Camões, nº57, 9004-514 Funchal, Portugal; anacelia.bett@gmail.com; 3 Serviço de Gastrenterologia, Hospital Dr. Nélio Mendonça, SESARAM, EPERAM, Avenida Luís de Camões, nº57, 9004-514 Funchal, Portugal; isabeljardim@sesaram.pt (I.J.); miguelluciocamacho@sesaram.pt (M.C.); 4 Departamento de Química, Faculdade de Ciências Exatas e Engenharia, Campus da Penteada, Universidade da Madeira, 9020-105 Funchal, Portugal

**Keywords:** colorectal cancer, urine, biomarkers, SPME/GC-MS, statistical analysis, exploratory study

## Abstract

Colorectal cancer (CRC) remains one of the leading causes of cancer-related mortality worldwide, largely due to delayed diagnosis. Current screening approaches are limited by invasiveness, cost, and low patient adherence, underscoring the urgent need for non-invasive biomarkers that could complement the existing strategies. Cancer-associated metabolic reprogramming, together with alterations in host–microbiota interactions, is reflected in the urinary volatilome, offering a potential source of candidate biomarkers. In this exploratory, case–control pilot study, urinary volatile organic metabolites (VOMs) were profiled in patients with colorectal cancer (CRC, *n* = 19) and healthy controls (HCs, *n* = 17) using headspace solid-phase microextraction coupled with gas chromatography–mass spectrometry (HS-SPME/GC–MS). Univariate and multivariate statistical modelling was applied to characterize disease-associated metabolic patterns. Sixty-seven urinary VOMs were identified, with terpenoids, ketones, phenolic compounds and norisoprenoids representing the predominant chemical classes. Following participant-level analysis and correction for multiple comparisons, 18 VOMs remained statistically significant between the two groups, consistent with metabolic perturbations previously associated with colorectal carcinogenesis, including gut microbial dysbiosis, oxidative stress, lipid peroxidation, chronic inflammation and altered energy metabolism. Orthogonal partial least squares-discriminant analysis (OPLS-DA), validated with participant-level cross-validation and 1000 permutations, revealed a separation between CRC and HC groups, supporting the existence of a disease-associated urinary volatilomic profile. Notably, one participant initially classified as an HC was subsequently diagnosed with metastatic CRC. This participant clustered with the CRC group in an unsupervised analysis performed using the original group label, without knowledge of the later diagnosis, raising the hypothesis, to be confirmed in a prospective cohort, that urinary volatilomic alterations may be detectable before clinical diagnosis. Our findings indicate that urinary volatilomic profiling captures metabolic changes associated with CRC and represents a promising, hypothesis-generating starting point for non-invasive biomarker discovery.

## Simple Summary

Colorectal cancer (CRC) is a major cause of cancer death worldwide, often because it is found too late. Current screening tests can be uncomfortable, expensive, or simply avoided by patients, so new, easier ways to detect this cancer are needed. This study looked at whether chemical compounds found in urine, produced naturally by the body and gut bacteria, could help identify people with colorectal cancer. Urine samples from 19 patients with CRC and 17 healthy controls (HCs) were analyzed using a specialized technique that detects these compounds. Sixty-seven compounds were identified, including terpenoids, ketones, phenolic compounds and norisoprenoids, and 18 of them reflected differences between the two groups associated with gut microbial alterations, oxidative stress, lipid peroxidation, chronic inflammation, and altered energy metabolism. These differences matched changes already known to occur in cancer, such as altered gut bacteria and increased cell stress. Using these compounds, a computer-based analysis could separate most cancer patients from healthy people. Interestingly, one person originally classified as healthy was later diagnosed with advanced colorectal cancer, and their urine pattern had already resembled that of cancer patients before this diagnosis was known. While this is only one case and needs further confirmation, it suggests that urine testing might one day help detect colorectal cancer earlier, in a simple and non-invasive way. Larger studies are still needed before this approach could be used in clinical practice.

## 1. Introduction

Colorectal cancer (CRC) remains one of the most significant oncological challenges of the 21st century, ranking as the third most common malignancy and the second leading cause of cancer-related mortality worldwide [1,2]. According to GLOBOCAN 2022 estimates, approximately 1,926,425 new CRC cases were diagnosed globally, accounting for 9.6% of all incident cancers, with 904,019 associated deaths representing 9.3% of cancer mortality. These facts are projected to worsen substantially by 2030, with rising incidence anticipated across all world regions, most markedly in Asia, Latin America, and Africa, driven by population ageing, urbanisation, and the adoption of Western dietary patterns [3,4,5].

**Figure 1:**
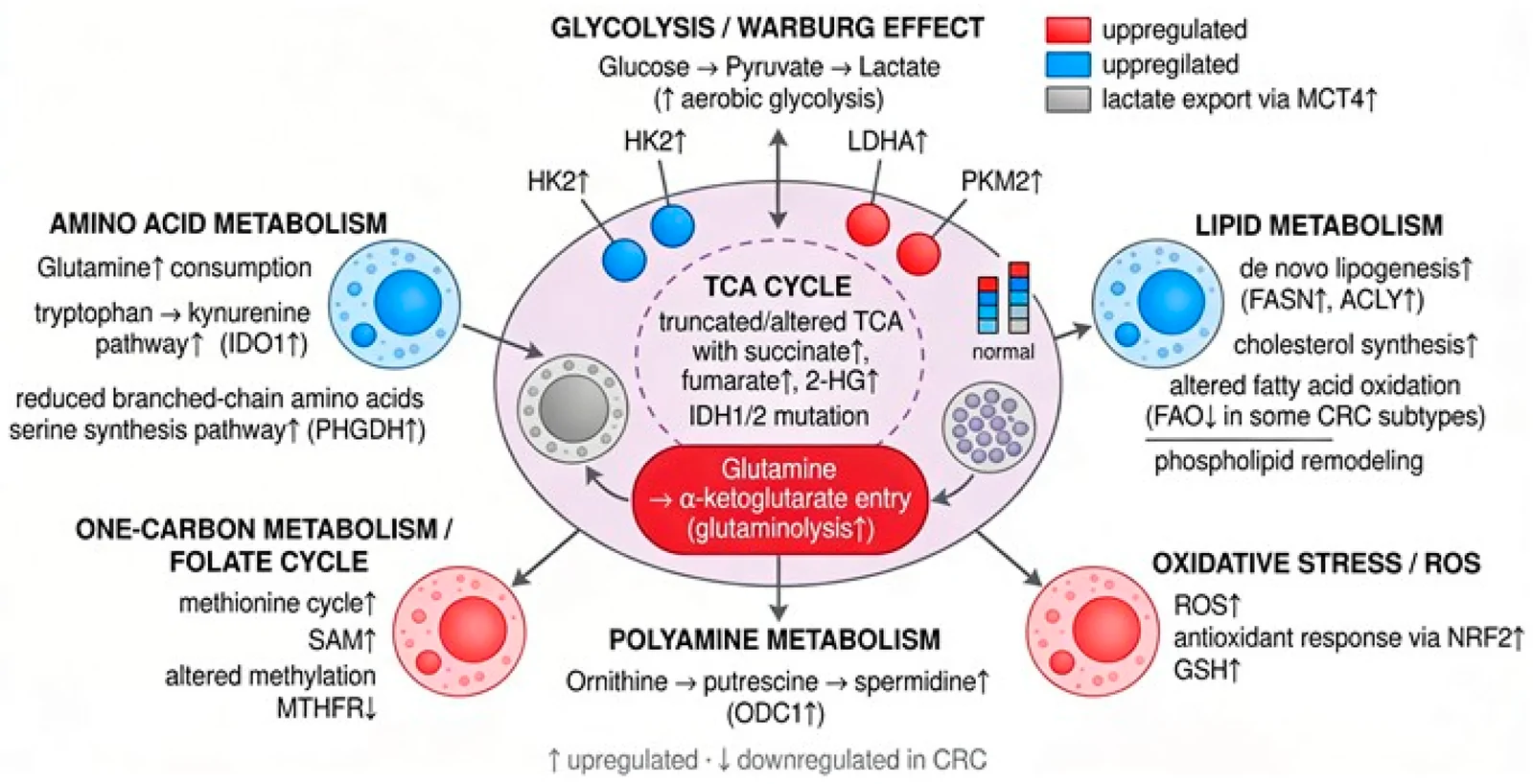
Main metabolic pathway alterations in CRC (based on [13,14,15]).

The carcinogenesis process encompasses a range of intricate mechanisms, including profound alterations in cellular biochemistry and a series of metabolic adaptations that are hallmarks of tumour development (Figure 1). CRC arises through a multistep adenoma-carcinoma sequence, involving sequential mutations (APC, KRAS, TP53) and DNA mismatch repair deficiency, and other signalling pathways that collectively promote metabolic reprogramming and tumour progression. Both environmental and hereditary factors contribute to colorectal carcinogenesis. Lifestyle-related factors, including obesity, unhealthy dietary habits, smoking, alcohol consumption, and physical inactivity, promote chronic inflammation, oxidative stress, insulin resistance, and metabolic dysregulation, thereby creating a pro-tumorigenic microenvironment. Genetic predisposition, particularly hereditary syndromes such as Lynch syndrome and familial adenomatous polyposis, further increases disease susceptibility through defects in DNA repair and tumour suppressor pathways. Together, these factors drive the metabolic reprogramming that characterizes CRC progression [6,7,8,9,10,11,12]. Despite considerable research efforts, many of these mechanisms remain incompletely understood, and their characterisation at the level of small metabolites is still largely insufficient. These biological alterations reshape cellular metabolism and host–microbiota interactions, leading to changes in the production of volatile metabolites (VOMs) that can be detected in urine and exploited as potential biomarkers of CRC.

The clinical course of CRC is heavily determined by the stage at diagnosis. Five-year survival rates exceed 90% for stage I disease but fall dramatically to below 10% for metastatic stage IV presentations. Yet CRC remains largely asymptomatic in its early stages, and a substantial proportion of patients are diagnosed only after the disease has advanced to loco-regional or metastatic spread, a reality that renders early detection not merely advantageous but imperative. Despite the proven efficacy of population-based screening programmes in reducing CRC incidence and mortality, current modalities face significant limitations in terms of sensitivity, patient adherence, and clinical accessibility.

Primary prevention focuses on modifications of lifestyles: the cessation of tobacco products, a Mediterranean-style diet, 150–300 min of weekly exercise, and chemoprevention (such as aspirin and COX-2 inhibition, as well as supplementation with calcium and folate) [16,17,18,19]. The gold standard for CRC screening—colonoscopy—offers the dual benefit of lesion detection and polypectomy, with studies demonstrating reductions in CRC incidence and mortality of 29–68%. However, its invasive nature, requirements for bowel preparation and sedation, and associated procedural risks contribute to suboptimal participation rates in population-based programmes. Non-invasive stool-based alternatives, such as the faecal immunochemical test (FIT) and the multitarget stool DNA test, achieve satisfactory sensitivity for established CRC (79–92%) but perform substantially worse for precancerous adenomas, limiting their impact on true primary prevention [17,18,20]. Blood-based assays, such as the SEPT9 methylation test, offer ease of collection but are constrained by their modest diagnostic performance, with a sensitivity of 68% and specificity of 80%, and remain outside mainstream screening guidelines. Taken together, these limitations sustain an urgent and unmet clinical need for non-invasive, cost-effective biomarkers capable of detecting CRC and its precursor lesions at the earliest possible stage [21,22].

In this context, endogenous VOMs have emerged as a particularly promising class of biomarkers. VOMs are low-molecular-weight compounds produced by both endogenous metabolic processes and the tumour microenvironment, and their profiles in biological fluids reflect the biochemical reprogramming associated with malignant transformation. Analysis of urinary VOMs is of particular interest: urine offers a non-invasive, easily repeatable, and logistically uncomplicated biological matrix that contains numerous metabolites at concentrations comparable to plasma, yet with reduced protein complexity and lower requirements for pre-analytical processing. The urinary volatilome has emerged as a promising approach for investigating tumour-associated metabolic alterations and identifying non-invasive biomarkers of colorectal cancer. Because urinary volatile organic metabolites originate from both host metabolism and gut microbial activity, they provide a unique window into the metabolic reprogramming, oxidative stress, inflammatory responses, and host–microbiota interactions that accompany colorectal carcinogenesis. Several classes of volatile metabolites, including ketones, phenolic compounds, sulphur-containing compounds, furans, and terpenoids, have been consistently associated with CRC, highlighting their biological relevance as potential disease biomarkers [1,20,23,24,25,26].

Seminal studies have already demonstrated the discriminatory potential of urinary VOM profiles for CRC detection. Cheng and colleagues [23] identified a seven-metabolite model comprising citric acid, hippuric acid, p-cresol, 2-aminobutyric acid, myristic acid, putrescine, and kynurenate that distinguished CRC patients from healthy controls with an area under the receiver operating characteristic curve (AUROC) of 0.993 and sensitivity and specificity exceeding 97% in the training set. Qiu et al. [27] further demonstrated that urinary metabolite profiles could differentiate CRC by disease stage, and that postoperative samples exhibited a partial return toward metabolic profiles observed in healthy individuals, suggesting that VOMs reflect tumour-driven systemic metabolic alterations rather than non-specific inflammatory responses. Complementary work employing field asymmetric ion mobility spectrometry has reported sensitivities of up to 88% and demonstrated that VOM signatures offer superior discriminatory performance over conventional faecal occult blood testing in bowel cancer screening contexts.

Despite these encouraging findings, the translation of urinary VOM-based diagnostics into clinical practice remains constrained by methodological heterogeneity, limited sample sizes, the absence of standardised pre-analytical protocols, and insufficient large-scale external validation. The field has yet to converge on a robust, reproducible biomarker panel validated across independent cohorts that is adequate for regulatory approval and clinical deployment. Against this background, the present study aimed to characterize the urinary volatilomic alterations associated with CRC and to identify biologically relevant VOMs reflecting tumour-associated metabolic reprogramming. By analysing urine samples from 19 histologically confirmed CRC patients and 17 healthy controls (HCs), we sought to improve understanding of disease-associated metabolic changes and to identify candidate biomarkers for non-invasive early detection.

## 2. Materials and Methods

### 2.1. Materials and Reagents

Sodium chloride (NaCl, 99.5%) was acquired from Panreac AppliChen ITW Reagents (Barcelona, Spain) to promote VOMs’ salting-out. Hydrochloric acid (HCl, 37%), 3-octanol (internal standard (IS), 99%), and C7 to C30 alkane solution were obtained from Sigma-Aldrich (St. Louis, MO, USA). The solutions of HCl 5 M and 3-octanol 5 parts per million (ppm) were prepared in ultrapure water obtained from a Milli-Q water purification system (Millipore, Bedford, PA, USA). The helium of purity 5.0 (Air Liquide, Algés, Portugal) was used as GC carrier gas. The glass vials, SPME holder for manual sampling, and fibre were purchased from Supelco (Merck KGaA, Darmstadt, Germany). The SPME device included a fused silica fibre coating partially cross-linked with 50/30 µm divinylbenzene/carboxen/polydimethylsiloxane (DVD/CAR/PMDS), which was conditioned at 270 °C for 30 min before its use, according to the manufacturer’s guidelines. Pure standards were used to confirm the VOMs identified through the National Institute of Standards and Technology (NIST) library and were acquired in their maximum available purity, which included 1-hexanol (98%), 2-ethyl-1-hexanol (98%), 2-methylbutanal (98%), 2-penthyl furan (98%), limonene (98%), nerolidol (98%), cadalene (98%), and 2-pentanone (98%) from Acros Organics (Fair Lawn, NJ, USA), dimethyl disulfide (>98%), hexanal (98%), α-ionone (98%), β-damascenone (98%), p-cymene (98%), linalool (98%), β-cyclocitral (98%), phenol (98%), p-cresol (98%), toluene (98%), butyl acetate (98%), and 4-heptanone (96%) from Fluka Analytical (Honeywell Specialty Chemicals Seelze GmbH, Hannover, Germany), and 3-hexanone (98%) from Aldrich Chemistry (St. Louis, MO, USA).

### 2.2. Subjects and Urine Sampling

This study represents an observational, case–control, cross-sectional pilot/feasibility study designed to characterize urinary volatilomic alterations associated with CRC and to identify candidate biomarkers for future non-invasive detection strategies. The study was not designed or powered as a diagnostic-accuracy or screening-validation study, and no a priori sample-size calculation was performed; the cohort size reflects the number of eligible, consenting participants recruited during the study period.

Urine of CRC patients and HCs was collected into 100 mL sterile, airtight polypropylene containers with an integrated transfer device (Greiner Bio-One GmbH, Kremsmünster, Austria) in the morning (without fasting) at Hospital Dr. Nélio Mendonça, SESARAM, EPERAM, Funchal. Samples were transported to the laboratory in a portable cooling box (±4 °C) suitable for biological specimens and processed within one hour of collection. Upon arrival, samples were centrifuged and aliquoted into 8 mL glass vials (to prevent repeated freeze–thaw cycles) and immediately stored at −80 °C until analysis, following the same procedure carried out with similar studies under development. Each aliquot was thawed only once before analysis. The study was performed in accordance with the principles contained in the Declaration of Helsinki and approved by the Ethical Committee for Health of SESARAM, EPERAM (S.24004958). All participants provided written informed consent prior to inclusion.

CRC patients were eligible if they had histologically confirmed colorectal adenocarcinoma. HCs were selected from blood donors of the Hospital Dr. Nélio Mendonça, SESARAM, EPERAM, Funchal, and were eligible if aged 40–60 years or older. The study included 19 CRC patients and 17 HCs (Table 1). Detailed individual clinicopathological characteristics, including tumor stage and TNM classification, and additional clinical characteristics, including cancer history, comorbidities, renal function, smoking status, and alcohol use, are provided in Supplementary Tables S1 and S2, respectively. All data collected from participants were processed to respect confidentiality, privacy, and the ethical principles inherent to research involving human subjects.

### 2.3. HS-SPME Procedure

The HS-SPME extraction was performed according to previously optimized conditions for the establishment of the urinary volatilomic signature [28]. Briefly, 4 mL aliquots of urine adjusted to pH 1–2 with 500 µL of HCl (5 M) were transferred to an 8 mL sampling glass vial, and 0.8 g of NaCl and 10 µL of 3-octanol (IS, 5 ppm) were added, with stirring at 800 rpm. The vial was placed in a thermostat bath adjusted to 50.0 ± 0.1 °C. Then, the DVD/CAR/PDMS fibre was inserted into the vial for 60 min. After sampling, the SPME fibre was withdrawn into the needle, removed from the vial, and inserted into the injector port (250 °C) of the GC-MS system for 6 min, for desorption of the analytes. Each sample was analysed in triplicate.

### 2.4. GC-MS Analysis

The GC-MS analysis was performed with an Agilent Technologies 6890N Network (Palo Alto, CA, USA). The gas chromatograph was equipped with a SUPELCOWAX 10 fused silica column (60 m  ×  0.25 mm I.D.  ×  0.25 μm film thickness, SGE, Dortmund, Germany). The chromatographic protocol selected for the separation of VOM prior to MS analysis consisted of setting the initial oven temperature at 40 °C for 2 min, followed by a temperature gradient of 2.7 °C/min up to 220 °C, which was maintained for 5 min, at a total run time of 73.67 min. The column flow was kept constant at 1 mL/min, using helium as the carrier gas (mobile phase). The injection port operated in splitless mode and was maintained at 250 °C. For the 5975 MS system, the transfer line, quadrupole, and source temperature were set at 270, 150, and 230 °C, respectively. The electron impact mass spectrum was acquired at an ionization energy of 70 eV and an emission current of 10 μA. Data acquisition was performed in scan mode (30–300 *m*/*z*), and the electron multiplier was adjusted using the auto-tune mode. The identification of VOMs was performed by comparing mass spectra with the data system library (NIST, 2005 software, Mass Spectral Search Program v. 2.2, Nist 2005, Gaithersburg, MD, USA), considering a minimum percentage match of 80%, Kovats index, and standard as available at the laboratory. To determine the Kovats index for the identified VOMs and allow their comparison with the Kovats index available in the literature for similar experimental conditions, the C7–C30 *n*-alkanes series was analysed under the same experimental conditions. A maximum difference of ΔKI ≤ 30 between the calculated and literature KI values was established as the acceptance criterion for retention-index agreement. All experiments were performed in triplicate, and the results were expressed as the mean relative peak area  ±  standard deviation.

### 2.5. Statistical Analysis

Statistical analysis was performed using MetaboAnalyst 6.0 [29]. Technical triplicates were used to assess analytical repeatability and were averaged for each participant prior to univariate and multivariate statistical analyses. Thus, each participant constituted a single independent biological observation in the final statistical matrix (CRC, *n* = 19; HC, *n* = 17; Supplementary Table S3). The workflow began with data pre-processing, which involved removing VOMs with missing values and normalizing the dataset. Normalization was carried out using cubic root transformation followed by auto-scaling.

For univariate analysis, differences in individual urinary VOM levels between CRC patients and HCs were assessed using Welch’s independent-samples *t*-test, which does not assume equal variances between groups. To account for multiple comparisons across the 67 evaluated VOMs, raw *p*-values were adjusted using the Benjamini–Hochberg false discovery rate (FDR) procedure. Statistical significance was defined as an FDR-adjusted *p*-value < 0.05. Effect sizes were estimated using Hedges’ g, together with 95% confidence intervals. Positive Hedges’ g values indicate higher levels in CRC patients, whereas negative values indicate higher levels in HCs.

Statistical analysis was performed using one-way analysis of variance (ANOVA) to assess differences in VOM abundance between the healthy control (HC) and colorectal cancer (CRC) groups. Where significant differences were detected, an appropriate post hoc multiple-comparison test was applied. A *p*-value < 0.05 was considered statistically significant. The statistical analysis was used to identify VOMs showing differential abundance between the two study groups and to support the selection of candidate metabolites for further biological and diagnostic evaluation. Multivariate statistical analysis was performed using participant-level mean relative peak areas. Principal component analysis (PCA) and orthogonal partial least squares-discriminant analysis (OPLS-DA) were performed using participant-level mean relative peak areas. PCA was conducted to uncover trends and detect potential outliers, followed by OPLS-DA. Key variables in the OPLS-DA model were assessed using variable importance in projection (VIP) scores higher than 1.4. Hierarchical clustering analysis based on Pearson’s correlation was used to generate a heatmap, helping to identify clustering patterns among significantly altered VOMs across the study groups. OPLS-DA model validation was conducted using participant-level cross-validation, ensuring complete separation of participants between training and validation subsets, together with 1000 permutation tests. Model performance was assessed using R^2^Y and Q^2^ values. As complementary exploratory measures of classification performance, accuracy, sensitivity, specificity, positive predictive value (PPV), negative predictive value (NPV), and the area under the receiver operating characteristic curve (AUROC) were calculated from participant-level cross-validated predictions. Ninety-five percent confidence intervals (95% CIs) were calculated using the Wilson method for proportions and the DeLong method for AUROC. These performance measures were considered exploratory given the limited sample size and the absence of an independent validation cohort.

## 3. Results and Discussion

### 3.1. Urinary Volatilomic Signature

**Figure 2:**
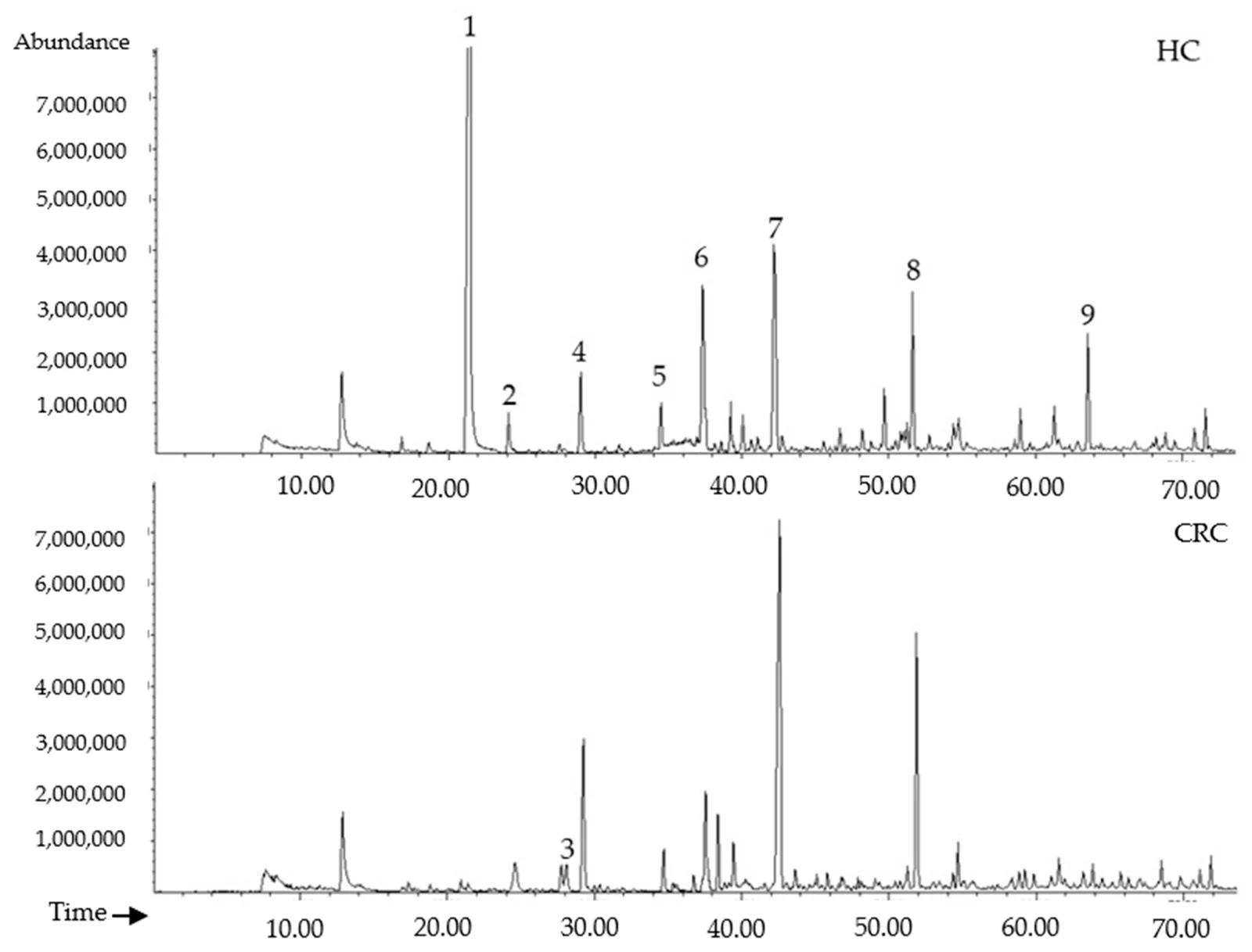
Representative chromatograms of the groups analyzed obtained using HS-SPME/GC-MS. Legend: HC: control group; CRC: colorectal group. peak number identification 1: 4-heptanone; 2: 2-heptanone; 3: γ-terpinene; 4 p-cymene; 5: 3-octanol (IS); 6: α-ocimene; 7: theaspirane; 8: 1,2-dihydro-1,1,6-trimethyl-naphthalene; 9: p-cresol.

Urinary volatilomic profiling revealed clear and reproducible differences between HC and CRC groups, indicating a distinct disease-associated metabolic profile, as shown in Figure 2.

A total of 67 VOMs were identified, spanning several chemical families, with ketones, norisoprenoids, and phenolic compounds representing the most abundant families. Several VOMs exhibited group-specific abundance patterns, while multivariate modelling confirmed a clear separation between the two cohorts.

**Figure 3:**
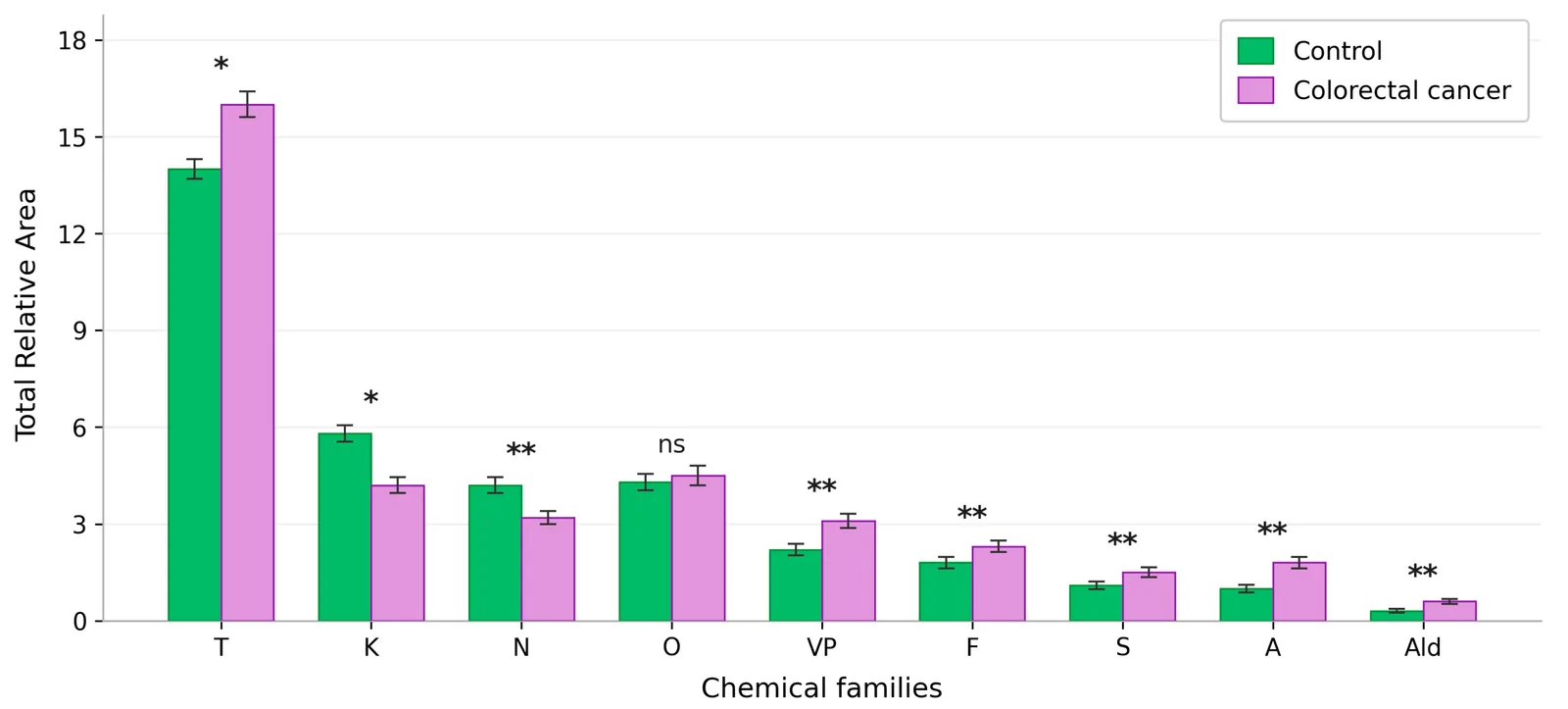
Total relative areas of chemical families identified in control (HC, *n* = 17) and colorectal cancer (CRC, *n* = 19). Legend: A: alcohols; Ald: aldehydes; F: furanic compounds; K: ketones; N: norisoprenoids; O: others; S: sulphur-containing compounds; T: terpenoids; VP: volatile phenols. Significance levels are indicated as * *p* < 0.05; ** *p* < 0.01; ns: not significant.

These alterations in the relative areas of these chemical families between HCs and CRC patients were also found to be significant, as determined by statistical analysis through Welch’s *t*-test. Aldehydes, alcohols, sulphur-containing compounds, furanic compounds, volatile phenols, and terpenoids were found to be significantly higher in CRC samples than in HCs, particularly aldehydes, which showed a clear increase (Figure 3). On the other hand, ketones and nitrogen-containing compounds showed a significant reduction in CRC samples, whereas there was no statistical difference in the total relative area of other VOMs between the two groups. The urinary volatilome is a complex system that is liable to change due to several factors, such as diet, stress, exercise, pharmacotherapy, and environmental influences that can impact the urinary volatilomic pattern. On the other hand, some changes can occur in the urine due to enzymatic activity, pH variations, bacterial action, and decomposition [30,31,32].

Terpenoids represent a structurally diverse class of VOMs contributing significantly to the urinary volatilomic profile and account for 40.2 and 40.5% of the total volatilomic profiles of HC and CRC groups, respectively. In humans, urinary terpenoids originate predominantly from exogenous sources, based on dietary consumption of terpene-rich food and beverages, as well as by exposure to cosmetics, perfumes, household cleansers, and pharmaceuticals containing essential oils [33,34]. Among the 25 terpenoids identified, only 11 terpenoids (e.g., γ-terpinene, p-cymene, α-ocimene, α-terpinyl acetate) were identified in both groups investigated, being their relative area higher in the HCs group (Table 2). The remaining terpenoids were only identified in the CRC group, such as γ-terpinene, limonene, linalool, and related oxides, previously associated with disease-related alterations in dietary patterns, gastrointestinal absorption, or metabolic processing [33]. Notably, p-cymene and γ-terpinene, though proposed as CRC-associated biomarkers [26], exhibited contrasting trends in this cohort.

Urinary ketones originate from both endogenous metabolic processes and exogenous sources. Endogenously, they arise mainly from carbohydrate metabolism and fatty acid β-oxidation, with acetone representing a principal product of lipid catabolism [26,35]. Additional contributions derive from gut microbiota activity, while dietary intake and environmental exposure constitute relevant exogenous sources [27]. Ketones with mixed endogenous and exogenous origins, including 3-hexanone, 4-heptanone, and 2-heptanone, were detected, alongside predominantly exogenous ketones such as 2-methyl-2-heptanone, and 3-octanone. These VOMs have also been associated with inflammatory and metabolic diseases, including celiac disease, ulcerative colitis, and Crohn’s disease [36,37]. Compared with HCs, CRC patients had higher relative areas of 2-pentanone and 3-hexanone, while lower levels of 4-heptanone and 2-heptanone. Notably, 2,3-pentadione, 2-methyl-2-heptanone, 3-octanone and 2-methylacetophenone were only detected in CRC groups (Table 2). These variations may reflect metabolic dysregulation associated with CRC and/or distinctive dietary, microbial or environmental exposures associated with CRC. Although both 2-pentanone and 4-heptanone have been put forward as urinary biomarkers for CRC, the opposite trends for these two compounds suggest the possible variability from disease stages, patient heterogeneity, or methodological factors [24].

Norisoprenoids were the third family that contributed the most to the urinary volatilomic profile of the HC and CRC groups, representing 12.8 and 9.61% of the total volatilomic profile, respectively, often of exogenous origin. These VOMs are formed by the enzymatic degradation of dietary carotenoids, often found in fruits [28]. TDN and β-damascenone were found to have lower relative area in the CRC patients (1.83 and 0.57, respectively) when compared to the HCs (2.79 and 0.93). These values were found to be reduced by almost twice in the case of CRC patients. TDN has earlier been considered a potential biological marker for CRC [26]. However, TDN levels have decreased in CRC patients compared to HCs in the present study. It could reflect alterations in various metabolic pathways associated with CRC or differences in the consumption, absorption, or bioavailability of precursor compounds from carotenoids. On the other hand, α-ionene was detected exclusively in the CRC group, raising the possibility of a link with exogenous exposures or with metabolic pathways altered during carcinogenesis. This observation is exploratory and would benefit from confirmation in an independent cohort.

Volatile phenols represent 6.60 and 7.57% of the total volatilomic profile of HC and CRC groups, respectively. These VOMs can arise from both endogenous and exogenous metabolic processes. p-Cresol is a gut microbiota-derived volatile metabolite primarily generated through the bacterial fermentation of tyrosine and, to a lesser extent, phenylalanine [26,38]. It has been extensively associated with gastrointestinal disorders, including inflammatory bowel diseases and CRC, and has also been purported as a microbial-derived biomarker in pathological conditions [36,37]. In the present study, p-cresol was detected at relatively high levels in both HC and CRC groups, with a moderate reduction observed in the CRC group, which may reflect disease-associated changes in gut microbial activity or substrate availability. Phenol, previously associated with microbial metabolism [39], showed increased abundance in CRC patients, which is consistent with previously published results, suggesting its potential as a candidate CRC-associated urinary biomarker. In conclusion, these results are consistent with a potential role of phenolic compounds as candidate biomarkers of microbiota alterations in CRC, although the multifactorial influences on microbial metabolism mean this association cannot be interpreted as causal or diagnostic without further validation [23,25,26,27].

Furanic compounds, largely of dietary origin, accounted for 4.61 and 5.41% of the volatilomic profile in HCs and CRC patients, respectively. These VOMs arise mainly from the thermal degradation of carbohydrates during food processing, with minor endogenous contributions linked to oxidative stress and lipid peroxidation. Several furans exhibited group-specific patterns: 2-methylfuran and 2,5-dimethylfuran were detected exclusively in CRC patients, whereas the mean relative area of 2-pentylfuran and 4,7-dimethylbenzofuran was reduced in CRC compared to HCs (Table 2). Although some furanic compounds have been associated with cancer in previous studies, their altered levels in CRC patients may reflect differences in dietary habits, oxidative stress status, and metabolic reprogramming associated with disease progression.

Sulphur-containing compounds exhibited significant differences between CRC patients and HCs, despite representing a relatively small proportion of the total urinary volatilome (2.96% in HCs and 4.50% in CRC patients). These VOMs mainly originate from the incomplete metabolism of sulfur-containing amino acids and are strongly modulated by gut microbiota activity [40]. Within this chemical family, dimethyl disulfide was detected in all samples from both groups, displaying comparable abundance ranges (0.05–1.22 in HCs and 0.12–1.25 in CRC). In contrast, 1,3-dithiane and 3-sulfolene were detected exclusively or predominantly in CRC patients, with 3-sulfolene present in 11% of CRC samples and absent in HCs. Dimethyl disulfide has been consistently reported as a candidate CRC-associated volatile biomarker, previously associated with alterations in sulphur metabolism and microbial dysbiosis. Its increased abundance in CRC patients in the present study is consistent with this prior candidacy, although its diagnostic relevance would need to be confirmed through formal sensitivity/specificity analysis in an independent cohort [24].

In addition to the major chemical families, a miscellaneous class of VOMs, which included alcohols, aldehydes, aromatic hydrocarbons, esters, lactones, and polycyclic compounds, also contributed to the urinary volatilome. Most of these VOMs were largely exogenous products that resulted from environmental exposure, diet, or drug use [41,42,43]. Hexanal, associated with lipid peroxidation, was detected only in HCs, whereas benzaldehyde was exclusive to CRC patients, suggesting disease-related alterations in oxidative stress and microbial metabolism [43,44]. These differences collectively point to considerable volatilomic changes, which highlight the discrimination capacity of VOMs as non-invasive biomarkers, thus validating the interest of using urine as a specimen for CRC diagnosis. In addition, the volatilome of urine is a complex system influenced not only by endogenous metabolism but also by exogenous effects, including dietary intake and drugs. To minimize inter-individual variability, urine was collected in the morning without a fasting requirement, consistent with real-world clinical conditions. The application of multivariate chemometric analysis helped distinguish between subjects based on consistent volatilomic signatures rather than individual metabolites.

### 3.2. Statistical Analysis and Identification of Discriminant Urinary VOMs

Statistical analysis was performed using MetaboAnalyst 6.0. Relative peak areas obtained by HS-SPME/GC–MS were normalized prior to statistical analysis to reduce systematic variability. Data preprocessing included filtering features with missing values, followed by cubic root transformation and autoscaling.

Univariate analysis identified 21 of the 67 urinary VOMs as nominally different between CRC patients and HCs (raw *p* < 0.05). Following Benjamini–Hochberg correction for multiple comparisons, 18 VOMs remained statistically significant (FDR-adjusted *p* < 0.05). These included 2,5-dimethylfuran, 2-pentanone, 2-ethyl-5-methylfuran, 3-hexanone, dimethyl disulfide, hexanal, limetol, octanal, hemimellitene, α-ocimene, 2-ethyl-1-hexanol, durene, theaspirane, ocimenol, naphthalene, β-damascenone, nerolidol, and cadalene. The corresponding raw *p*-values, FDR-adjusted *p*-values, Hedges’ g effect sizes, and 95% confidence intervals are reported in Supplementary Table S4.

Subsequently, one-way ANOVA with post hoc testing was applied, considering *p*-values < 0.05 as statistically significant. These tests identified significant differences between the VOMs of the HC group and CRC patient group for 51 out of the 67 identified VOMs. It is also noteworthy that some of these identified VOMs include 2-pentanone, 3-hexanone, dimethyl disulfide, p-cymene, 2-methylfuran, and 4-heptanone. These have been previously identified as CRC-associated urinary volatilome and include metabolomic processes such as microbial dysbiosis, lipid peroxidation, and intestinal inflammation [24,26,27,35]. Other VOMs such as α-terpinyl acetate, cosmene, β-damascenone, and 3-sulfolene also became interesting candidate biomarkers based on significant differential abundance and universal presence in CRC specimens, although these require further evaluation for diagnostic potential. Conversely, metabolites mainly linked with environmental exposures, like *p*-xylene or naphthalene, presented evidence of alterations but possess less likelihood for specific biomarker status [35,36,37].

**Figure 4:**
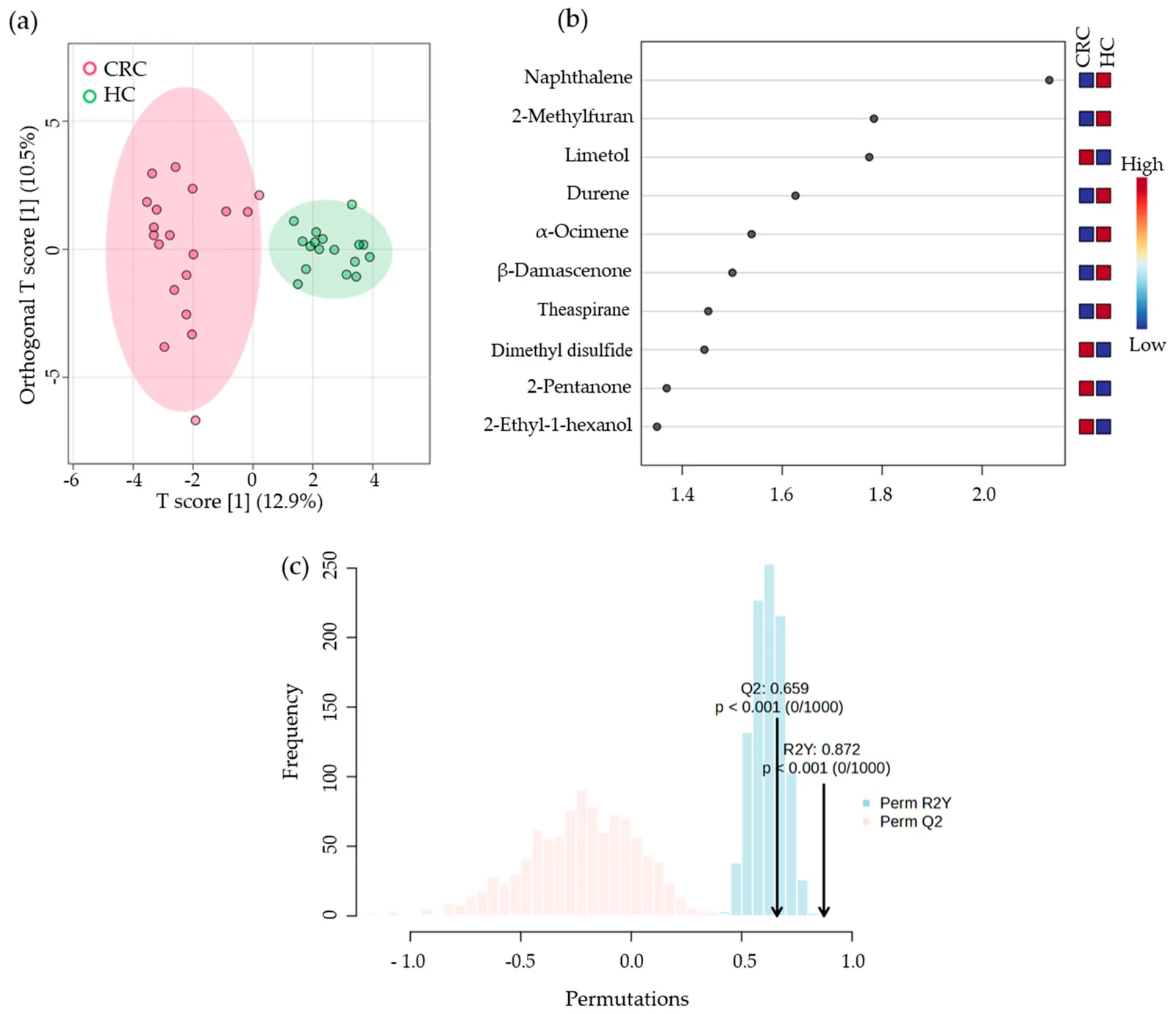
(**a**) Orthogonal partial least squares-discriminant analysis (OPLS-DA) scores plot; (**b**) variables of importance in projection (VIP) scores plot; (**c**) OPLS-DA model validation by permutation tests based on 1000 random permutations of the VOMs obtained by GC-MS of the urine samples from the groups under study. T score [1] is the score of each sample on the model’s first predictive component, that is, the linear combination of variables (here, the measured volatile compounds) that best separates the two groups (CRC vs. HC).

OPLS-DA demonstrated a clear separation between CRC patients and HC, consistent with the existence of a distinct disease-associated urinary volatilomic profile (Figure 4a). The VIP score plot (Figure 4b) identified the VOMs contributing most significantly to group discrimination in the multivariate model. The metabolites that indicated VIP values > 1.4 were designated as having significant influence. The VOMs that exhibited the highest discriminative properties among these VOMs were naphthalene, 2-methylfuran, limetol, durene, α-ocimene, β-damascenone, theaspirane, dimethyl disulfide, 2-pentanone, and 2-ethyl-1-hexanol. Notably, limetol, dimethyl disulfide, 2-pentanone, and 2-ethyl-1-hexanol were predominantly associated with CRC patients, whereas the remaining VOMs were more closely associated with HCs. The discriminant VOMs comprise metabolites of both endogenous and exogenous origin, several of which have been previously linked to CRC, intestinal inflammation, Crohn’s disease, microbial metabolic activity, and lipid peroxidation processes. Their elevated VIP values underscore their strong contribution to the multivariate classification model and support their relevance as potential non-invasive biomarkers for CRC.

**Figure 5:**
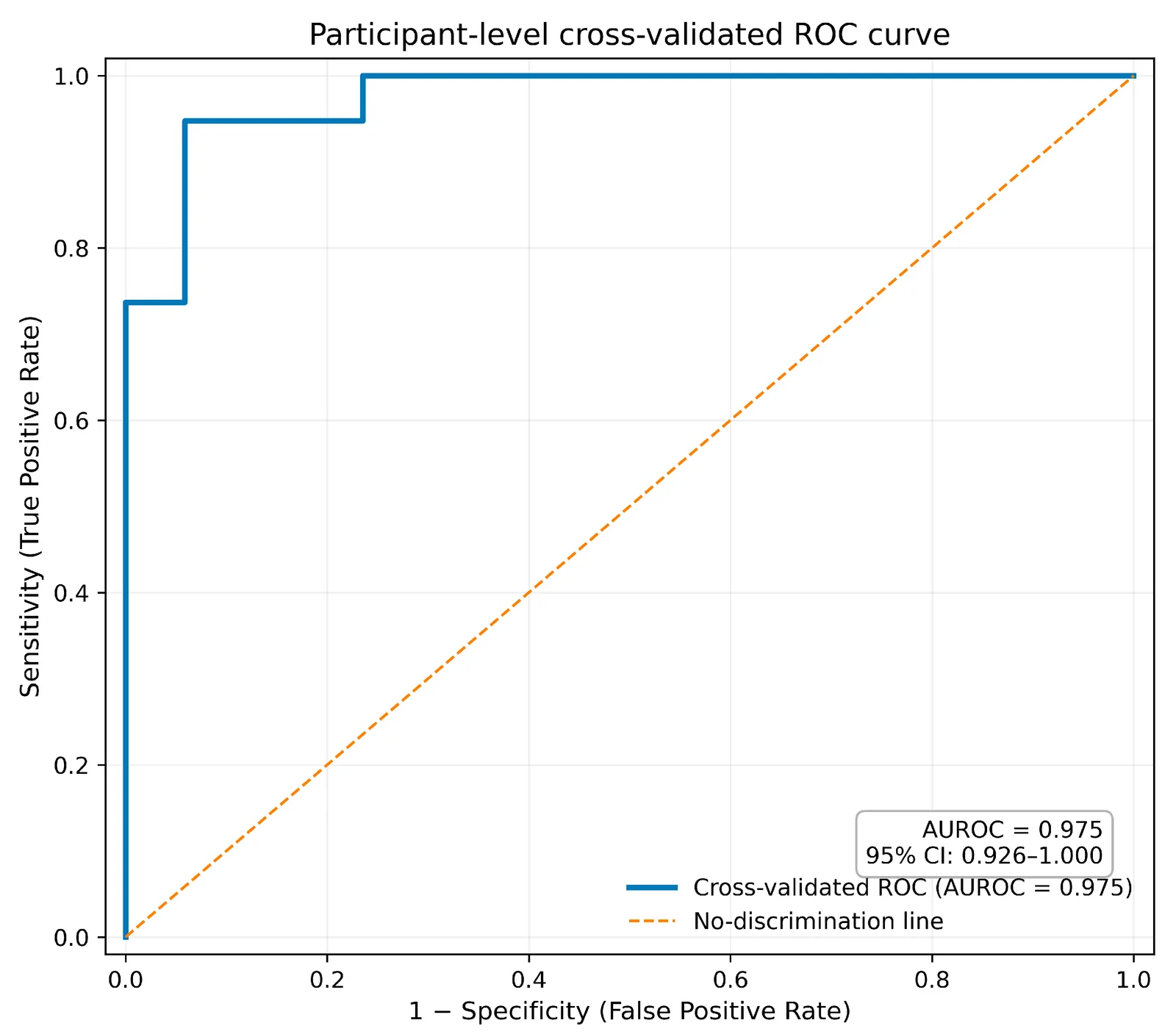
Receiver operating characteristic (ROC) curve based on participant-level cross-validated predictions for discrimination between colorectal cancer (CRC) patients and healthy controls (HCs).

Permutation testing confirmed the robustness of the OPLS-DA model, as none of the 1000 permuted models produced R^2^Y or Q^2^ values exceeding those of the original model (Figure 4c). The revised model yielded R^2^Y = 0.872 and Q^2^ = 0.659. Model stability was further evaluated using 1000 permutation tests, in which the permuted models showed lower R^2^Y and Q^2^ values than the original model. To assess model stability and reduce the risk of overfitting associated with supervised classification methods, the OPLS-DA model was evaluated using participant-level stratified 7-fold cross-validation and 1000 permutation tests. None of the permuted models achieved a Q^2^ value equal to or greater than that of the original model (maximum permuted Q^2^ = 0.357; permutation *p* = 0.001), supporting that the observed discrimination was unlikely to have arisen by chance. Participant-level stratified 7-fold cross-validation yielded an accuracy of 91.7% (95% CI: 78.2–97.1%), sensitivity of 84.2% (95% CI: 62.4–94.5%), specificity of 94.1% (95% CI: 73.0–99.0%), positive predictive value (PPV) of 94.1% (95% CI: 74.2–99.0%), and negative predictive value (NPV) of 84.2% (95% CI: 62.4–94.5%). The cross-validated AUROC was 0.975 (95% CI: 0.926–1.000) (Figure 5). Despite these high exploratory classification estimates, they should be interpreted cautiously given the small cohort size and absence of an independent validation cohort.

**Figure 6:**
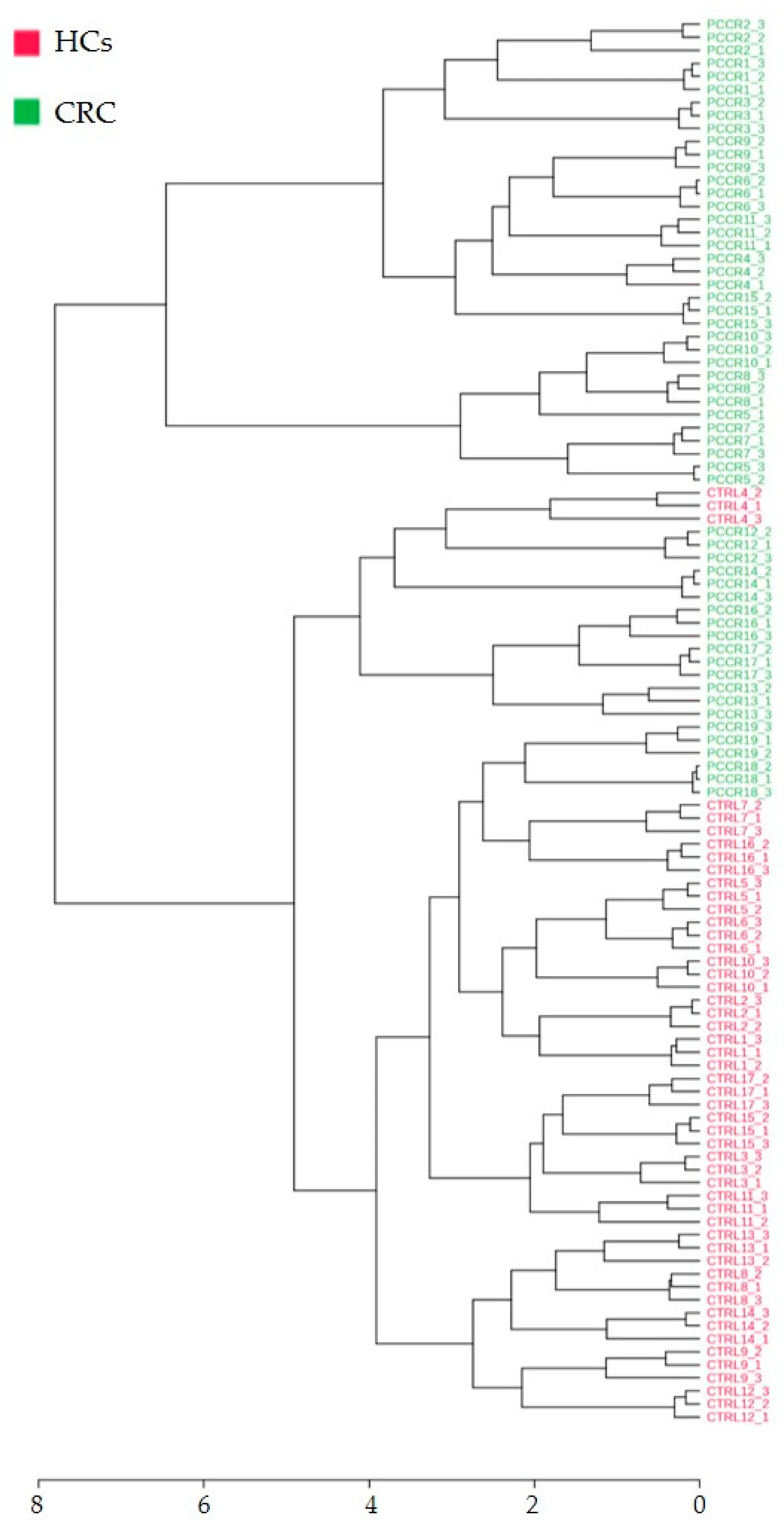
Hierarchical clustering dendrogram of urinary volatilomic profiles from controls (HCs = CTRL) and colorectal cancer patients (CRC).

These findings support the presence of systematic differences in urinary volatilomic profiles between CRC patients and HCs, while acknowledging the exploratory nature and limited sample size of the study. A hierarchical clustering analysis (dendrogram) based on the urinary volatilomic profile revealed a distinct grouping pattern between the HC and CRC groups (Figure 6).

However, one individual initially enrolled and labelled as a healthy control (CTRL4) clustered with the CRC group in both the unsupervised hierarchical clustering analysis and the OPLS-DA scores plot. Both analyses were performed using this participant’s original group label, without knowledge of any subsequent diagnosis. Four months after urine collection, this participant was diagnosed with metastatic CRC. At the time of urine collection, the participant had suspected symptoms. This single retrospective observation raises the hypothesis that urinary volatilomic alterations may be detectable before clinical diagnosis of CRC. However, because this participant was ultimately found to have metastatic disease, the finding cannot be interpreted as evidence that the assay detects CRC at an early, pre-symptomatic stage, nor can a single case establish the discriminative power of the model. This observation is presented as hypothesis-generating and warrants prospective confirmation in a dedicated, adequately powered cohort of at-risk individuals followed over time.

Given the limited sample size, multivariable adjustment for multiple demographic and clinical covariates was not performed to avoid model overfitting and unstable estimates. These potential confounding effects could be assessed in larger independent cohorts.

## 4. Conclusions

This study shows that CRC is associated with a distinct urinary volatilomic profile consistent with metabolic alterations previously linked to tumor development and progression. A total of 67 VOMs were identified in urine samples from 19 CRC patients and 17 HCs. Following participant-level analysis and correction for multiple comparisons, 18 VOMs remained statistically significant between the two groups. Several discriminative VOMs, including 2-pentanone, octanal, dimethyl disulfide, 2-ethyl-1-hexanol and 2-methylfuran, have previously been associated with biological processes implicated in colorectal carcinogenesis, such as gut microbial dysbiosis, oxidative stress, lipid peroxidation, chronic inflammation, and altered cellular metabolism. These associations remain to be confirmed by direct biological measurement in future work.

Multivariate chemometric analysis, validated at participant level, revealed a urinary volatilomic profile that separated CRC patients from HCs. One participant initially classified as a HC was subsequently diagnosed with metastatic CRC and had already clustered with the CRC group in an unsupervised, label-blind analysis. While compatible with volatilomic alterations preceding clinical diagnosis, this is a single retrospective case and should be regarded as hypothesis-generating rather than as evidence of an early-detection capability.

Given the exploratory design, limited sample size, and the absence of externally validated diagnostic-accuracy metrics in the present analysis, these findings should be interpreted as a candidate urinary volatilomic profile rather than a validated diagnostic tool. This work represents a first step toward a non-invasive urinary test for CRC detection. The next steps in this line of research include: (i) validation of the candidate metabolite panel in larger, independent, prospective, multi-centre cohorts with a priori sample-size calculation; (ii) targeted, quantitative confirmation of the top discriminant VOMs using authentic standards; (iii) formal evaluation of diagnostic performance (sensitivity, specificity, AUROC) once a validated panel is defined, including assessment across tumour stages to test early-detection capability; (iv) integration with complementary multi-omics data, particularly gut microbiome and metabolomics profiling, to clarify the biological origin of the discriminant VOMs and improve biomarker specificity.

## Figures and Tables

**Table 1:** Demographic and clinical characteristics of participants in the control (HC) and colorectal cancer (CRC) groups.

Variable	HC (*n* = 17)	CRC (*n* = 19)
**Sex [*n* (%)]**		
Male	13 (76.5%)	15 (78.9%)
Female	4 (23.5%)	4 (21.1%)
**Mean Age ± SD (years)**	68.7 ± 12.1	66.9 ± 11.9
**Smoking status [*n* (%)]**		
Ever smokers	5 (29.4%)	7 (36.8%)
Never smokers	12 (70.6%)	12 (63.2%)
**Tumor topography [*n* (%)]**		
Cecum	n/a	2 (10.5%)
Ascending colon		2 (10.5%)
Sigmoid colon		5 (26.3%)
Rectosigmoid junction		2 (10.5%)
Rectum		8 (42.1%)
**Tumor morphology [*n* (%)]**		
Adenocarcinoma	n/a	19 (100%)
**Stage at diagnosis [*n* (%)]**		
I	n/a	2 (10.5%)
II	n/a	7 (36.8%)
III	n/a	6 (31.6%)
IV	n/a	3 (15.8%)
Unknown	n/a	1 (5.3%)

Data are presented as absolute numbers and percentages [*n* (%)] or as mean ± standard deviation (SD), where applicable. Tumor topography, morphology and stage at diagnosis were provided only for colorectal (CRC) patients. Abbreviations: n/a—not applicable.

**Table 2:** Volatile organic metabolites, chemical family, possible origin, and range of relative peak area of the identified VOMs in the HC group and CRC patients (*n* = 3; RSD < 20%).

RT (min)	VOMs	KI_cal_	KI_Lit_	Possible Origin	Frequency (%)		Range RPA	
					HC	CRC	HC	CRC
**Alcohols**								
32.2	1-Hexanol *	1299	1308	Exo	-	16	-	0.52–0.77
39.6	2-Ethyl-1-hexanol *	1451	1453	Endo/Exo	100	100	0.46–1.95	0.81–3.80
**Aldehydes**								
15.6	2-Methylbutanal *	972	949	Exo	-	16	-	0.33–0.51
19.0	Hexanal *	1050	1063	Endo/Exo	47	-	0.09–0.88	-
30.2	Octanal	1265	1263	Exo	-	42	-	0.11–0.92
41.8	Benzaldehyde	1494	1490	Endo/Exo	-	16	-	0.14–0.83
**Furanic Compounds**								
11.5	2-Methylfuran	866	870	Endo/Exo	-	16	-	0.12–0.50
14.1	2,5-Dimethylfuran	936	946	Exo	-	58	-	0.02–2.63
17.3	2-Ethyl-5-methylfuran	1014	1018	Exo	100	32	0.06–1.18	0.11–1.25
27.2	2-Pentylfuran *	1208	1213	Exo	71	42	0.18–1.35	0.12–0.62
50.2	4,7-Dimethylbenzofuran	1680	1677	Exo	71	79	0.12–1.73	0.13–0.86
**Ketones**								
14.9	2-Pentanone *	957	974	Exo	12	79	0.23–0.34	0.09–2.35
18.0	3-Hexanone *	1030	1046	Endo/Exo	18	58	0.03–0.05	0.03–0.19
18.2	2,3-Pentadione	1037	1044	Exo	-	21	-	0.02–0.89
21.7	4-Heptanone *	1104	1103	Endo/Exo	100	100	0.20–40.7	0.18–8.33
24.6	2-Heptanone	1161	1151	Endo/Exo	35	21	0.03–0.97	0.03–0.15
25.7	2-Methyl-2-heptanone	1181	1178	Exo	-	5	-	0.27
28.3	3-Octanone	1230	1224	Exo	-	5	-	0.44
52.8	2-Methylacetophenone	1743	1738	Endo/Exo	-	5	-	0.29
**Norisoprenoids**								
41.5	Theaspirane	1489	1496	Exo	100	58	0.11–1.79	0.13–0.93
43.8	α-Ionene *	1534	1545	Exo	-	11	-	0.26–0.68
46.0	β-Ionene	1585	1605	Exo	18	32	0.16–0.55	0.17–0.91
52.1	TDN	1723	1722	Exo	100	100	0.38–7.88	0.12–7.68
54.4	β-Damascenone *	1780	1791	Exo	100	79	0.16–3.66	0.14–1.37
**Sulphur-containing compounds**								
18.6	Dimethyl disulfide *	1042	1044	Endo/Exo	100	100	0.05–1.22	0.12–1.25
29.8	1,3-Dithiane	1259	1276	Exo	6	5	0.15	0.22
31.2	Butyl isothiocyanate	1283	1294	Exo	94	89	0.12–0.94	0.13–0.85
51.0	3-Sulfolene	1680	-	Exo	-	11	-	0.72–0.73
**Terpenoids**								
21.1	Limetol	1093	1096	Exo	-	58	-	0.04–0.38
23.2	(*E*)-β-ocimene	1234	1242	Exo	-	11	-	0.20–0.30
25.4	Eucalyptol	1176	1179	Exo	-	11	-	0.24–0.32
25.8	Limonene *	1183	1195	Exo	-	21	-	0.24–0.81
27.8	Ocimene quintoxide	1219	1215	Exo	12	26	0.21–0.31	0.12–0.39
28.1	γ-Terpinene	1226	1233	Exo	6	11	3.53	0.76–1.36
29.5	p-Cymene *	1252	1233	Exo	100	89	0.20–7.05	0.16–4.57
36.9	Tetrahydrolinalool	1393	1397	Exo	35	58	0.16–0.94	0.12–1.54
37.8	α-Ocimene	1410	1404	Exo	100	58	0.32–29.8	0.19–5.48
38.6	Dihydromyrcerol	1427	1439	Exo	76	84	0.11–2.30	0.14–10.0
39.1	Linalool oxide	1438	1440	Exo	65	58	0.10–0.80	0.11–1.95
39.3	Cosmene	1444	1446	Exo	-	26	-	0.11–0.94
40.3	(*Z*)-Limonene oxide	1464	1467	Exo	-	5	-	0.57
42.3	Linalool *	1505	1506	Exo	-	11	-	0.13–0.26
46.4	β-Cyclocitral *	1591	1598	Exo	-	5	-	0.63
46.8	Menthol	1600	1599	Exo	63	67	0.12–1.23	0.16–0.77
48.1	Ocimenol	1630	1617	Exo	-	47	-	0.17–0.84
49.3	*α*-Terpinyl acetate	1658	1679	Exo	6	42	0.61	0.25–0.72
50.2	Borneol	1679	1677	Exo	-	5	-	0.24
51.3	Carvotanacetone	1704	1697	Exo	-	16	-	0.54–1.87
53.3	Cuminaldehyde	1752	1751	Exo	12	11	0.28–0.88	0.82–0.86
62.8	Nerolidol *	2074	2076	Exo	-	37	-	0.11–0.65
68.5	δ-Cadinol	2147	2134	Exo	12	11	0.16–0.18	0.26–2.45
70.0	Cadalene *	2165	2178	Exo	100	84	0.15–0.71	0.10–0.56
71.3	Eudesmol	2197	2205	Exo	100	100	0.21–0.89	0.16–1.56
**Volatile phenols**								
60.0	2-Bromophenol	2002	2011	Endo/Exo	-	5	-	0.79
61.3	Phenol *	2035	2035	Exo	65	95	0.12–1.16	0.10–2.63
64.1	p-Cresol *	2104	2103	Endo/Exo	100	79	0.46–6.53	0.28–7.64
68.7	Eugenol	2163	2157	Exo	-	11	-	0.14–0.25
**Others**								
17.6	Toluene *	1021	1027	Exo	-	11	-	0.25–0.36
20.5	Butyl acetate *	1082	1075	Exo	-	5	-	0.02
22.6	p-Xylene	1123	1130	Exo	6	11	0.09	0.08–0.33
32.8	Hemimellitene	1314	1329	Endo/Exo	88	58	0.12–1.00	0.14–0.76
40.5	Durene	1468	1463	Exo	100	68	0.35–1.26	0.14–1.13
48.3	Lavender lactone	1636	1635	Exo	-	11	-	0.18–0.79
50.6	Ethyl hydroxyhexanoate	1682	1677	Endo/Exo	-	11	-	0.48–0.78
51.7	Naphthalene	1715	1713	Exo	100	42	0.24–2.97	0.14–1.86
61.8	Butyl laurate	2048	2035	Exo	94	95	0.14–2.29	0.10–2.72
62.1	2,6-Dimethylnaphthalene	2056	2038	Exo	24	11	0.15–0.70	0.25–0.32

Abbreviation: Exo: exogenous; Endo: endogenous; HC: control group; CRC: colorectal cancer; RT: retention time; KI_Cal_: kovat index calculated; KI_Lit_: kovat index from the literature for equivalent columns; TDN: 1,2-dihydro-1,1,6-trimethyl-naphthalene. Maximum ΔKI accepted between calculated and literature values is ≤30; * the VOM was confirmed against authentic standard.

## Data Availability

The original contributions of this study are fully included in the article. Any additional inquiries should be addressed to the corresponding authors. Supplementary Table S1. Clinicopathological characteristics and TNM staging of individual tumor samples included in the colorectal cancer (CRC) group; Supplementary Table S2. Clinical history, comorbidities, renal function, and lifestyle characteristics of individual patients included in the colorectal cancer (CRC) group; Supplementary Table S3. Participant-level dataset used for univariate and multivariate statistical analyses of urinary volatile organic metabolites (VOMs) in colorectal cancer (CRC) patients and healthy controls (HCs). Technical triplicates were averaged for each participant prior to statistical analysis; Supplementary Table S4. Participant-level univariate analysis of 67 urinary VOMs in CRC patients (*n* = 19) and HCs (*n* = 17). Technical triplicates were averaged before analysis. Between-group differences were assessed using Welch’s *t*-test with Benjamini–Hochberg FDR correction. Effect sizes are reported as Hedges’ g with 95% CIs.

## Supplementary Materials

The following supporting information can be downloaded at: https://www.mdpi.com/article/10.3390/biology15181578/s1, Table S1: Clinicopathological characteristics and TNM staging of individual tumor samples included in the colorectal cancer (CRC) group; Table S2: Clinical history, comorbidities, renal function, and lifestyle characteristics of individual patients included in the colorectal cancer (CRC) group; Table S3: Data matrix used in the Statistical treatment; Table S4: Raw *p*-values, FDR-adjusted *p*-values, Hedges’ g effect sizes, and 95% confidence intervals.

## Abbreviations

The following abbreviations are used in this manuscript:

APC	adenomatous polyposis coli
AUROC	area under the receiver operating characteristic curve
BRAF	B-Raf proto-oncogene, serine/threonine kinase
CI	confidence interval
COPD	chronic obstructive pulmonary disease
COX-2	cyclooxygenase-2
CRC	colorectal cancer
DVD/CAR/PMDS	divinylbenzene/carboxen/polydimethylsiloxane
FDR	false discovery rate
FIT	faecal immunochemical test
GC-MS	gas chromatography mass spectrometry
HCs	healthy controls
HS-SPME	headspace solid-phase microextraction
KRAS	Kirsten rat sarcoma viral oncogene homolog
MMR	mismatch repair
MSI	microsatellite instability
NF-κB	nuclear factor kappa-light-chain-enhancer of activated B cells
NPV	negative predictive value
OPLS-DA	orthogonal partial least squares-discriminant analysis
PCA	principal component analysis
PPV	positive predictive value
SD	standard deviation
TDN	1,2-dihydro-1,1,6-trimethyl-naphthalene
TP53	tumour protein 53
VIP	variables of importance in projection
VOMs	volatile organic metabolites
