# Supplementary material for: Urinary Volatilomic Profiling Reveals Candidate Metabolomic Signatures Associated with Colorectal Cancer
Source: Biology (Basel). 2026 Sep 8;15(18):1578. doi: 10.3390/biology15181578 (PMC13603885; doi:10.3390/biology15181578)
Supplement: Supplementary file 1 [file biology-15-01578-s001.zip › biology-4500889-supplementary.pdf]

Table S1

Clinicopathological characteristics and TNM staging of individual tumor samples included in the colorectal cancer (CRC) group.

Sample	Sex	Topography	Morphology	Stage at diagnosis	cTNM [a]	pTNM [b]
**1**	Male	Rectum	Adenocarcinoma	IIIB	T3N2M0	ypT0N0M(n/a)
**2**	Male	Rectum	Adenocarcinoma	IIIB	T3N1M0	ypT0N0M(n/a)
**3**	Male	Rectum	Adenocarcinoma	IIIB	T3N1M0	ypT3N0M0
**4**	Male	Rectosigmoid junction	Adenocarcinoma	IIA	T3N0M0	T3N0M0
**5**	Male	Ascending colon	Adenocarcinoma	IIIB	TxNxM0	T3N1aM(n/a)
**6**	Female	Rectum	Adenocarcinoma	IIA	T3N0M0	-
**7**	Male	Sigmoid colon	Adenocarcinoma	IVA	T3N2M1a	-
**8**	Female	Ascending colon	Adenocarcinoma	IV	T4bN2M1	T4aN2bM(n/a)
**9**	Male	Cecum	Adenocarcinoma	I	TxN0M0	T2N0M(n/a)
**10**	Female	Rectum	Adenocarcinoma	Unknown (refused further examinations)	TxN0M0	-
**11**	Male	Rectum	Adenocarcinoma	IVA	TxN1M1a	-
**12**	Female	Sigmoid colon	Adenocarcinoma	I	TxN0M0	T2N0M(n/a)
**13**	Male	Rectum	Adenocarcinoma	IIA	T3N0M0	ypT0N0M(n/a)
**14**	Male	Sigmoid colon	Adenocarcinoma	IIIA	TxN0M0	T1N1aM(n/a)
**15**	Male	Sigmoid colon	Adenocarcinoma	IIA	T3NxM0	T3N0M(n/a)
**16**	Male	Sigmoid colon	Adenocarcinoma	IIA	TxN0M0	T3N0M(n/a)
**17**	Male	Rectum	Adenocarcinoma	IIIB	T3N2aM0	ypT2N0M(n/a)
**18**	Male	Cecum	Adenocarcinoma	IIA	TxN0M0	T3N0M(n/a)
**19**	Male	Rectosigmoid junction	Adenocarcinoma	IIA	TxN0M0	T3N0M(n/a)

[a] Clinical TNM classification – determined by clinical exams, imaging, and biopsies before surgery.

[b] Pathological TNM – determined by surgical removal and histopathological microscopic analysis. All urine samples were collected before any CRC-directed treatment; pTNM reflects subsequent pathological assessment; and "yp" prefix indicates TNM classification after neoadjuvant therapy and surgical resection.

Abbreviations: n/a – not applicable; TNM – tumor node metastasis staging.

Table S2

Clinical history, comorbidities, renal function, and lifestyle characteristics of individual patients included in the colorectal cancer (CRC) group.

Sample	Cancer history [a]	Major comorbidities [a]	Renal function [a]		Smoking status [b]	Alcohol use [b]
			Creatinine (mg/dL)	eGFR (mL/min/1.73m^2)		
**1**	Bladder *in situ* tumor (Transitional cell carcinoma *in situ*) in remission for about 30 months	Diabetes	0.93	≥60	Ever smoker	No
**2**	n/d	n/d	0.83	≥60	Ever smoker	Yes
**3**	n/d	n/d	0.74	≥60	Ever smoker	Yes
**4**	Lung cancer (adenocarcinoma) diagnosed about 1 month after the sample collection	n/d	0.69	≥60	Ever smoker	No
**5**	n/d	Benign prostatic hyperplasia; dementia; hypertension	1.19	≥60	Ever smoker	No
**6**	Concurrent ascending colon *in situ* tumor (adenocarcinoma *in situ*)	Dementia; heart failure and hypertension	0.61	≥60	Never smoker	No
**7**	n/d	n/d	0.75	≥60	Never smoker	No
**8**	n/d	n/d	0.71	≥60	Never smoker	No
**9**	n/d	Diabetes and hypertension	1.08	≥60	Never smoker	No
**10**	n/d	Diabetes and heart failure	0.60	≥60	Never smoker	No
**11**	Non-Hodgkin Lymphoma diagnosed about 9 months prior to the sample collection	COPD; dyslipidemia; hypertension and heart failure	1.00	≥60	Ever smoker	Yes
**12**	n/d	Diabetes and hypertension	0.68	≥60	Never smoker	No
**13**	n/d	n/d	0.80	≥60	Ever smoker	Yes
**14**	n/d	Dyslipidemia and hypertension	0.84	≥60	Never smoker	Yes
**15**	n/d	Heart failure and hypertension	1.13	≥60	Never smoker	No
**16**	n/d	Diabetes; hypertension and purpura	0.88	≥60	Never smoker	Yes
**17**	n/d	n/d	0.76	≥60	Never smoker	No
**18**	n/d	Dyslipidemia and hypertension	0.74	≥60	Never smoker	No
**19**	n/d	Dyslipidemia and hypertension	1.04	≥60	Never smoker	No

[a] Data obtained through medical abstraction.

[b] Data obtained through patient questionnaire.

All CRC urine samples were collected before any CRC-directed treatment. No recent antibiotic use or major dietary restrictions were documented in the clinical records before or at the time of urine collection. Abbreviations: eGFR – estimated glomerular filtration rate; COPD – chronic obstructive pulmonary disease; n/d – not documented.

SUPPLEMENTARY TABLE S3

	VOM	2-Methylfu	2,5-Dimeth	2-Pentanon	2-Methylbu	2-Ethyl-5-n	Toluene	3-Hexanon
Samples	Group	1	2	3	4	5	6	7
PCCR1	CRC	0.12	0.05	0.5	0	0.18	0.25	0.03
PCCR2	CRC	0.5	0.6	0.3	0.51	0	0	0.05
PCCR3	CRC	0.22	2.63	2.35	0.33	0	0	0.19
PCCR4	CRC	0	0.16	0.65	0	0	0	0.07
PCCR5	CRC	0	0.02	0.14	0	0	0	0.01
PCCR6	CRC	0	0.04	0.14	0	0.11	0.36	0.01
PCCR7	CRC	0	0.05	0.26	0.47	0	0	0.05
PCCR8	CRC	0	0.09	0.33	0	0	0	0
PCCR9	CRC	0	0.07	0.17	0	0	0	0
PCCR10	CRC	0	0	0.25	0	0	0	0
PCCR11	CRC	0	0.1	0	0	1.25	0	0
PCCR12	CRC	0	0	0	0	0.22	0	0
PCCR13	CRC	0	0	0.07	0	0	0	0.09
PCCR14	CRC	0	0	0	0	0	0	0
PCCR15	CRC	0	0.18	0	0	0	0	0.17
PCCR16	CRC	0	0	0.09	0	0	0	0.06
PCCR17	CRC	0	0	0.36	0	0	0	0.06
PCCR18	CRC	0	0	0.39	0	0	0	0
PCCR19	CRC	0	0	0.18	0	0.15	0	0
CTRL1	HC	0	0	0	0	0.1	0	0
CTRL2	HC	0	0	0	0	0.08	0	0
CTRL3	HC	0	0	0	0	0.16	0	0
CTRL4	HC	0	0	0.34	0	0.41	0	0.05
CTRL5	HC	0	0	0	0	0.63	0	0
CTRL6	HC	0	0	0	0	0.42	0	0
CTRL7	HC	0	0	0.23	0	0.14	0	0.05
CTRL8	HC	0	0	0	0	0.52	0	0
CTRL9	HC	0	0	0	0	0.47	0	0
CTRL10	HC	0	0	0	0	0.06	0	0
CTRL11	HC	0	0	0	0	0.06	0	0
CTRL12	HC	0	0	0	0	0.22	0	0
CTRL13	HC	0	0	0	0	0.36	0	0
CTRL14	HC	0	0	0	0	0.15	0	0
CTRL15	HC	0	0	0	0	1.18	0	0
CTRL16	HC	0	0	0	0	0.37	0	0.03
CTRL17	HC	0	0	0	0	0.17	0	0

2,3-Pentad	Dimethyl d	Hexanal	Butyl aceta	Limetol	4-Heptano	p-Xylene	(E)-2-ocim	2-Heptano
8	9	10	11	12	13	14	15	16
0.02	0.65	0	0	0.04	0.72	0	0	0
0	0.25	0	0	0.22	4.89	0	0	0
0.89	0.14	0	0.02	0	3.4	0.08	0	0.12
0.07	0.12	0	0	0.11	1	0.11	0	0.08
0.03	0.05	0	0	0	0.83	0	0	0
0	1.15	0	0	0.06	2.86	0	0	0.03
0	0.46	0	0	0.21	0.97	0	0.2	0
0	0.29	0	0	0.08	5.95	0	0	0.15
0	1.25	0	0	0.38	2.46	0	0.3	0
0	0.66	0	0	0	4.81	0	0	0
0	0.33	0	0	0.16	0.33	0.33	0	0
0	0.47	0	0	0	0.21	0	0	0
0	0.14	0	0	0.8	2.46	0	0	0
0	0.19	0	0	0.09	0.18	0	0	0
0	0.68	0	0	0.12	2.42	0	0	0
0	0.21	0	0	0	1.31	0	0	0
0	0.08	0	0	0	8.33	0	0	0
0	0.13	0	0	0	1.8	0	0	0
0	0.32	0	0	0	1.23	0	0	0
0	0.09	0.09	0	0	2	0	0	0
0	0.09	0	0	0	6.42	0	0	0.13
0	0.17	0	0	0	1.3	0	0	0
0	0.11	0	0	0	0.67	0	0	0.03
0	0.13	0	0	0	40.71	0	0	0.56
0	0.17	0.12	0	0	33.34	0	0	0.97
0	0.07	0	0	0	3.96	0	0	0
0	1.22	0	0	0	2.63	0	0	0
0	0.1	0.13	0	0	3.06	0	0	0.09
0	0.1	0	0	0	0.18	0	0	0
0	0.1	0.88	0	0	1.64	0	0	0
0	0.17	0	0	0	0.2	0.09	0	0
0	0.05	0.15	0	0	2.73	0	0	0
0	0.08	0.06	0	0	2.43	0	0	0.05
0	0.1	0	0	0	4.54	0	0	0
0	0.06	0.04	0	0	2.07	0	0	0
0	0.08	0	0	0	1.33	0	0	0

Eucalyptol	2-Methyl-2	Limonene	2-Pentylfur	Ocimene q	a-Terpinen	3-Octanon	p-Cymene	1,3-Dithian
17	18	19	20	21	22	23	24	25
0.24	0	0.24	0	0	0	0	0.16	0
0	0	0.68	0.62	0	0	0	1.34	0
0.32	0.27	0	0	0	0	0	0	0.22
0	0	0	0	0	0	0.44	0.29	0
0	0	0	0	0	0	0	1.36	0
0	0	0	0	0	0	0	0.36	0
0	0	0	0.12	0	0	0	0.47	0
0	0	0	0.45	0	0	0	0.22	0
0	0	0	0.52	0	0	0	2.89	0
0	0	0	0	0	0	0	0	0
0	0	0.81	0.25	0	0	0	0.3	0
0	0	0	0	0	0	0	0.11	0
0	0	0	0.57	0	0	0	0.34	0
0	0	0	0	0.39	0.76	0	4.57	0
0	0	0	0	0.23	0	0	0.84	0
0	0	0	0.23	0.27	0	0	1.13	0
0	0	0	0.33	0.12	1.36	0	3.68	0
0	0	0	0	0	0	0	1.64	0
0	0	0	0	0.21	0	0	3.83	0
0	0	0	0.56	0	0	0	0.2	0
0	0	0	0.53	0.21	0	0	0.63	0
0	0	0	0.5	0	0	0	0.32	0
0	0	0	0	0	3.53	0	0.48	0.15
0	0	0	1.35	0	0	0	2.12	0
0	0	0	0.5	0	0	0	2.38	0
0	0	0	1.22	0	0	0	4.98	0
0	0	0	0.18	0	0	0	1.22	0
0	0	0	0.54	0	0	0	7.05	0
0	0	0	0.37	0.31	0	0	1.62	0
0	0	0	0	0	0	0	3.29	0
0	0	0	0.87	0	0	0	1.64	0
0	0	0	0	0	0	0	0.54	0
0	0	0	0.68	0	0	0	0.47	0
0	0	0	0	0	0	0	1.09	0
0	0	0	0.35	0	0	0	1.11	0
0	0	0	0	0	0	0	0.93	0

Octanal	Butyl isothi	1-Hexanol	Hemimellit	Tetrahydro	?-Ocimene	Dihydromy	Linalool ox	Cosmene
26	27	28	29	30	31	32	33	34
0	0.13	0	0.39	0.48	0	0.61	1.95	0.33
0	0	0	0	0	1.92	0.38	0.85	0
0.21	0.34	0	0.14	0.17	1.36	0.39	0.12	0
0	0.14	0	0.37	0.82	0	0.43	0	0
0.16	0.54	0	0	0.16	0.58	0.46	0.25	0
0.58	0.85	0	0.23	1.54	0	0	0.4	0.27
0.92	0.46	0.52	0	0.39	0	10.04	0	0
0	0.3	0	0	0	0	0.18	0	0
0.11	0.55	0	0.14	0	2.89	3.76	0.14	0.94
0	0.85	0	0	0.12	0	0.3	0	0
0	0.27	0	0	0.14	0	0.17	0.8	0
0	0.34	0	0	0	0.19	0	0	0
0	0.17	0	0.14	0.71	0.58	0.25	0.79	0.47
0.18	0	0	0.31	0.4	3.25	1.83	0	0.11
0.14	0.38	0.77	0.76	0	5.48	1.4	1.08	0
0	0.17	0	0.29	0	1.52	4.65	0	0
0	0.24	0	0.3	0	1.57	0.19	0	0
0	0.31	0	0.38	0.37	0	0	0.2	0
0	0.26	0	0	0	4.3	0.24	0.11	0
0	0.12	0	0.27	0.67	0.54	0.71	0.61	0
0	0.25	0	0.69	0	0.79	0	0.11	0
0	0.13	0	0	0	29.82	2.3	0	0
0	0.11	0	0.34	0	5.34	0.06	0.46	0
0	0.21	0	0.82	0.21	2.73	0.46	0.2	0
0	0.13	0	0.95	0.94	3.62	0.12	0	0
0	0.12	0	0.37	0.1	6.21	0.55	0	0
0	0.73	0	0.46	0	0.7	0	0	0
0	0.43	0	0.3	0.17	9.94	0	0	0
0	0.18	0	1	0.16	1.14	0	0.12	0
0	0.22	0	0.99	0	4.55	0.67	0.22	0
0	0	0	0.12	0	30.53	0.28	0.19	0
0	0.12	0	0.59	0	2.52	0.11	0.8	0
0	0.13	0	0.39	0	0.32	0.11	0.1	0
0	0.27	0	0	0	1.96	0	0.14	0
0	0.94	0	0.47	0	8.16	0.21	0	0
0	0.18	0	0.77	0	5.12	0.33	0	0

2-Ethyl-1-h	(Z)-limoner	Durene	Theaspiran	Benzaldehy	linalool	a-Ionene	b-Ionene	a-Cyclocitr
35	36	37	38	39	40	41	42	43
0.86	0	0.99	0.16	0	0	0.68	0.6	0
1.02	0	0	0.17	0	0.26	0	0	0
1.35	0.57	0.14	0	0.57	0	0	0.69	0
0.99	0	0.2	0	0	0	0	0	0
2.14	0	0	0	0	0	0	0	0
1.36	0	0.97	0.35	0.14	0.13	0	0.91	0
1.56	0	0.7	0	0	0	0	0	0
1.28	0	0	0	0	0	0	0	0
1.33	0	0.63	0	0	0	0	0.2	0.63
1.25	0	0	0	0.83	0	0	0	0
1.74	0	0.96	0.36	0	0	0	0.51	0
1	0	0	0.1	0	0	0.26	0	0
1.1	0	0	0.71	0	0	0	0	0
0.99	0	0	0	0	0	0	0	0
3.8	0	0.48	0.25	0	0	0	0	0
0.85	0	0.2	0.42	0	0	0	0	0
0.87	0	0.29	0.36	0	0	0	0	0
0.8	0	1.13	0.55	0	0	0	0.17	0
1.09	0	0.79	0.93	0	0	0	0	0
0.64	0	0.35	0.88	0	0	0	0	0
1.16	0	0.73	0.17	0	0	0	0.23	0
1.03	0	0.76	0.45	0	0	0	0	0
1.95	0	0.55	0.67	0	0	0	0	0
1.2	0	1.26	0.43	0	0	0	0	0
1.01	0	0.95	0.33	0	0	0	0	0
1.01	0	0.57	0.33	0	0	0	0	0
0.46	0	0.53	0.71	0	0	0	0	0
0.81	0	0.47	1.79	0	0	0	0	0
0.77	0	0.83	0.3	0	0	0	0.16	0
0.91	0	0.86	0.11	0	0	0	0	0
1.01	0	0.91	0.27	0	0	0	0.55	0
0.63	0	0.49	0.26	0	0	0	0	0
0.96	0	0.52	0.14	0	0	0	0	0
0.74	0	0.94	1.08	0	0	0	0	0
0.7	0	0.54	0.15	0	0	0	0	0
0.64	0	0.63	0.05	0	0	0	0	0

Menthol	Ocimenol	Lavender la	?-Terpinyl a	4,7-Dimeth	Borneol	Ethyl hydro	3-Sulfolene	Carvotanac
44	45	46	47	48	49	50	51	52
0.22	0	0.79	0	0	0.24	0	0	0
0.44	0.67	0	0.25	0.21	0	0	0.73	0
0	0	0.18	0.33	0.33	0	0.48	0.72	0.54
0	0	0	0	0.77	0	0	0	0
0.28	0.32	0	0	0.86	0	0	0	0
0	0.22	0	0	0.17	0	0	0	0
0	0	0	0.49	0.54	0	0.78	0	0
0	0	0	0	0	0	0	0	0
0.58	0.17	0	0.54	0.63	0	0	0	0
0.71	0	0	0	0	0	0	0	0
0	0.84	0	0.72	0.6	0	0	0	0
0	0	0	0	0	0	0	0	0
0.56	0	0	0	0.29	0	0	0	0
0.14	0.24	0	0.26	0	0	0	0	0
0.41	0.19	0	0.58	0.34	0	0	0	1.87
0.52	0	0	0	0.13	0	0	0	0
0.16	0	0	0	0.16	0	0	0	0
0.21	0	0	0	0.6	0	0	0	0
0.77	0	0	0	0.81	0	0	0	0
0.03	0	0	0	0.83	0	0	0	0
0	0	0	0	0.12	0	0	0	0
0.54	0	0	0	1.16	0	0	0	0
0	0	0	0.61	0.12	0	0	0	0
0.46	0	0	0	0.37	0	0	0	0
0.97	0	0	0	1.54	0	0	0	0
0	0	0	0	0	0	0	0	0
0.48	0	0	0	0	0	0	0	0
	0	0	0	0.38	0	0	0	0
0.24	0	0	0	1.73	0	0	0	0
0.44	0	0	0	0	0	0	0	0
0	0	0	0	0.27	0	0	0	0
0	0	0	0	0	0	0	0	0
0	0	0	0	0	0	0	0	0
1.23	0	0	0	0.37	0	0	0	0
0.6	0	0	0	0.94	0	0	0	0
0.12	0	0	0	0.48	0	0	0	0

Naphthaler	TDN	2-Methylac	Cuminalde	?-Damasce	2-Bromoph	Phenol	Butyl laurat	2,6-Dimeth
53	54	55	56	57	58	59	60	61
0	0.79	0	0.82	0.08	0		0.52	0
0.27	0.26	0	0.86	0.28	0.79	0.12	0.1	0
0.42	1.13	0	0	0	0	0.22	0.24	0
0	2.57	0.29	0	0.14	0	0.18	0.17	0.32
0	0.03	0	0	0	0	0.09	0.03	0
0	0.21	0	0	0.38	0	0.04	0.31	0
0.27	0.12	0	0	0	0	0.43	0.42	0.25
0	0.45	0	0	0	0	1.19	0.2	0
0	5.31	0	0	1.37	0	0.17	2.72	0
0	1.02	0	0	0.07	0	0.13	0.81	0
0	0.53	0	0	0	0	0.1	0	0
0	3.42	0	0	0.52	0	0.41	1.41	0
0	0.63	0	0	0.1	0	0.09	0.19	0
0	7.68	0	0	0.93	0	0.28	0.67	0
0.71	5.08	0	0	0.58	0	0.25	1.59	0
0.14	0.35	0	0	0.14	0	0.47	0.35	0
0.21	0.68	0	0	0.2	0	0.12	0.26	0
0	1.67	0	0	0.65	0	2.63	1.1	0
1.86	4.8	0	0	0.51	0	0.24	0.43	0
0.24	0.38	0	0	0.22	0	0.12	0.27	0
0.91	3.39	0	0	1.47	0	0.23	1.2	0.15
0.56	5.34	0	0	1.55	0	0	0.44	0
0.48	0.59	0	0	0.56	0	0.86	0.14	0.7
0.95	1.62	0	0	1.08	0	0.29	0.81	0
0.56	4.07	0	0	0.4	0	0.12	0.87	0
0.61	0.86	0	0.28	0.42	0	1.16	0.58	0
0.33	1.04	0	0.88	0.52	0	0.18	0.92	0
0.54	1.14	0	0	0.79	0	0	0.23	0
0.57	3.52	0	0	0.79	0	0.18	0.74	0
0.73	1.95	0	0	0.34	0	0.21	0.33	0
0.69	6.38	0	0	1.83	0	0	0	0
0.81	2.37	0	0	0.91	0	0	1	0
2.15	1.51	0	0	0.16	0	0	0.24	0
2.97	7.88	0	0	3.66	0	0	2.29	0
0.45	1.27	0	0	0.3	0	0.16	0.35	0
1.12	2.92	0	0	0.26	0	0.18	0.53	0

Nerolidol	p-Cresol	?-Cadinol	Eugenol	Cadalene	Eudesmol
62	63	64	65	66	67
0	0.34	0	0.14	0.1	0.3
0	0.28	0	0	0.23	0.28
0.11	0.41	0	0	0.11	0.28
0	3.37	0.26	0.25	0.4	0.28
0	0.65	0	0	0.03	0.16
0	0.4	0	0	0.05	0.22
0	0	0	0	0	0.28
0	1.45	0	0	0	0.2
0	0	0	0	0.46	0.33
0	0	0	0	0.03	0.18
0.12	5.28	0	0	0.12	0.28
0	7.64	2.45	0	0	1.56
0.65	1.38	0	0	0.15	0.33
0.17	0.61	0	0	0.39	0.4
0.19	3.28	0	0	0.28	0.57
0.45	0.74	0	0	0.56	0.32
0	1.66	0	0	0.11	0.44
0	2.21	0	0	0.29	0.22
0	0.48	0	0	0.31	0.48
0	0.65	0	0	0.21	0.27
0	4.79	0.16	0	0.28	0.62
0	1.07	0	0	0.3	0.45
0	2.13	0.18	0	0.15	0.21
0	3.94	0	0	0.18	0.8
0	2.79	0	0	0.19	0.49
0	1.16	0	0	0.44	0.41
0	1.56	0	0	0.71	0.27
0	0.58	0	0	0.37	0.4
0	0.46	0	0	0.3	0.52
0	2.02	0	0	0.15	0.49
0	0.53	0	0	0.3	0.44
0	1.9	0	0	0.37	0.54
0	0.81	0	0	0.34	0.44
0	6.53	0	0	0.61	0.89
0	1.13	0	0	0.15	0.3
0	1.03	0	0	0.19	0.63

SUPPLEMETARY TABLE S4

No.	VOM	CRC mean (cube-root)	CRC SD	HC mean (cube-root)
2	2,5-Dimethylfuran	0,316	0,364	0,000
3	2-Pentanone	0,530	0,342	0,077
5	2-Ethyl-5-methylfuran	0,171	0,316	0,638
7	3-Hexanone	0,224	0,214	0,062
9	Dimethyl disulfide	0,682	0,200	0,500
10	Hexanal	0,000	0,000	0,216
12	Limetol	0,315	0,303	0,000
26	Octanal	0,237	0,337	0,000
29	Hemimellitene	0,382	0,346	0,709
31	α-Ocimene	0,695	0,665	1,603
35	2-Ethyl-1-hexanol	1,081	0,149	0,961
37	Durene	0,517	0,431	0,878
38	Theaspirane	0,408	0,376	0,722
45	Ocimenol	0,256	0,355	0,000
53	Naphthalene	0,278	0,399	0,908
57	β-Damascenone	0,510	0,363	0,892
62	Nerolidol	0,197	0,309	0,000
66	Cadalene	0,479	0,260	0,659

HC SD	Mean difference (CRC-HC)	Difference 95% CI low	Difference 95% CI high	Welch t
0,000	0,316	0,141	0,491	3,789
0,218	0,453	0,260	0,646	4,788
0,184	-0,467	-0,641	-0,293	-5,479
0,138	0,162	0,041	0,283	2,730
0,156	0,182	0,061	0,303	3,065
0,294	-0,216	-0,367	-0,065	-3,033
0,000	0,315	0,169	0,460	4,534
0,000	0,237	0,075	0,399	3,072
0,301	-0,327	-0,546	-0,108	-3,031
0,712	-0,909	-1,377	-0,440	-3,943
0,108	0,120	0,032	0,208	2,776
0,097	-0,361	-0,573	-0,149	-3,553
0,217	-0,314	-0,520	-0,108	-3,110
0,000	0,256	0,084	0,427	3,137
0,202	-0,630	-0,843	-0,418	-6,075
0,262	-0,382	-0,595	-0,169	-3,644
0,000	0,197	0,048	0,345	2,776
0,108	-0,180	-0,314	-0,045	-2,758

Welch df	Raw p	BH-FDR adjusted p	Hedges' g	Hedges' g 95% CI low
18,000	0,001	0,013	1,168	0,471
30,894	0,000	0,001	1,526	0,790
29,484	0,000	0,000	-1,739	-2,501
31,036	0,010	0,043	0,870	0,198
33,425	0,004	0,028	0,987	0,305
16,000	0,008	0,038	-1,048	-1,735
18,000	0,000	0,004	1,398	0,677
18,000	0,007	0,034	0,947	0,269
33,979	0,005	0,028	-0,982	-1,663
32,919	0,000	0,005	-1,292	-2,002
32,677	0,009	0,040	0,890	0,216
20,018	0,002	0,017	-1,101	-1,793
29,340	0,004	0,028	-0,986	-1,668
18,000	0,006	0,032	0,967	0,287
27,270	0,000	0,000	-1,917	-2,703
32,638	0,001	0,010	-1,168	-1,866
18,000	0,012	0,046	0,856	0,184
24,545	0,011	0,043	-0,865	-1,537

Hedges' g 95% CI high	Raw p < 0.05	FDR < 0.05	Direction
1,866	Yes	Yes	Higher in CRC
2,261	Yes	Yes	Higher in CRC
-0,977	Yes	Yes	Higher in HC
1,543	Yes	Yes	Higher in CRC
1,668	Yes	Yes	Higher in CRC
-0,362	Yes	Yes	Higher in HC
2,119	Yes	Yes	Higher in CRC
1,625	Yes	Yes	Higher in CRC
-0,301	Yes	Yes	Higher in HC
-0,582	Yes	Yes	Higher in HC
1,564	Yes	Yes	Higher in CRC
-0,410	Yes	Yes	Higher in HC
-0,305	Yes	Yes	Higher in HC
1,647	Yes	Yes	Higher in CRC
-1,132	Yes	Yes	Higher in HC
-0,471	Yes	Yes	Higher in HC
1,527	Yes	Yes	Higher in CRC
-0,193	Yes	Yes	Higher in HC
